# Extragonadal FSHR Expression and Function—Is It Real?

**DOI:** 10.3389/fendo.2019.00032

**Published:** 2019-02-04

**Authors:** Marcin Chrusciel, Donata Ponikwicka-Tyszko, Slawomir Wolczynski, Ilpo Huhtaniemi, Nafis A. Rahman

**Affiliations:** ^1^Institute of Biomedicine, Research Centre for Integrative Physiology and Pharmacology, University of Turku, Turku, Finland; ^2^Institute of Animal Reproduction and Food Research, Polish Academy of Sciences, Olsztyn, Poland; ^3^Department of Reproduction and Gynecological Endocrinology, Medical University of Bialystok, Bialystok, Poland; ^4^Institute of Reproductive and Developmental Biology, Imperial College London, London, United Kingdom

**Keywords:** FSH, FSHR, extragonadal expression, cancer cells, tumor vessel cells

## Abstract

Expression of the follicle-stimulating hormone receptor (FSHR), besides gonadal tissues, has recently been detected in several extragonadal normal and tumorous tissues, including different types of primary and metastatic cancer and tumor vessel endothelial cells (TVEC). The suggested FSH actions in extragonadal tissues include promotion of angiogenesis, myometrial contractility, skeletal integrity, and adipose tissue accumulation. Non-malignant cells within cancer tissue have been shown to be devoid of FSHR expression, which implies a potential role of FSHR as a diagnostic, prognostic, or even a therapeutic tool. There are shared issues between several of the published reports questioning the validity of some of the conclusion. Firstly, protein expression of FSHR was performed solely with immunohistochemistry (IHC) using either an unavailable “in house” FSHR323 monoclonal antibody or poorly validated polyclonal antibodies, usually without additional methodological quality control and confirmations. Secondly, there is discrepancy between the hardly traceable or absent *FSHR* gene amplification/transcript data and non-reciprocal strong FSHR protein immunoreactivity. Thirdly, the pharmacological high doses of recombinant FSH used in *in vitro* studies also jeopardizes the physiological or pathophysiological meaning of the findings. We performed in this review a critical analysis of the results presenting extragonadal expression of FSHR and FSH action, and provide a rationale for the validation of the reported results using additional more accurate and sensitive supplemental methods, including *in vivo* models and proper positive and negative controls.

## Introduction

Follicle-stimulating hormone (FSH) is synthesized by the anterior pituitary gonadotroph cells, and it plays a critical role in controlling male and female gonadal function (1, 2). FSH acts through its specific receptor (FSHR), a member of the highly conserved family of class A G-protein-coupled receptors (GPCR) (3). In females, FSHR is expressed in granulosa cells and it regulates the maturation of Graafian follicles, granulosa cell proliferation and estrogen production (4). In males, FSHR is expressed in testicular Sertoli cells and it regulates their metabolic functions necessary for proper spermatogenesis and germ cell survival (5). FSHR activation may trigger a number of intracellular signaling pathways that will be activated in parallel or sequentially (6, 7). The canonical Gsα/cAMP/PKA signaling pathway, a key effector mechanism of FSH action, activates the cAMP response element-binding protein that modulates gene transcription (6, 7). However, in recent years, it has been shown that also Gαs-independent pathways, such as the PI3K/PIP3–AKT/mTOR pathway, β-arrestin-dependent pathway or interaction of FSHR with PPL1, FoxO1a, and 14-3-3τ, are involved in FSH-dependent cellular responses [reviewed in (8)].

Recent studies have suggested the expression of FSHR in many normal extragonadal tissues, as well as in the tumors and tumor vessel endothelial cells (TVECs) (summarized in **Table 1B**). Considering these new findings on extragonadal FSHR expression, a potential role for FSHR as a diagnostic, prognostic and therapeutic tool has been suggested. However, when some of the extragonadal FSHR findings have been revisited no *FSHR* transcript (by *in situ* hybridization RNAscope study) or gene amplification (by TaqMan probes-based qPCR) has been found in the tissues presenting with FSHR protein immunoreactivity by IHC staining (9, 10, 16, 17).

This review will summarize and discuss the most important extragonadal FSHR expression findings and highlights some of the caveats involved in these data. We offer our remarks in constructive spirit, and hope they will be found useful in future research on the intriguing topic of extragonadal FSH/FSHR action.

## Discrepancy Between FSHR mRNA and Protein Expression Levels

Surprisingly, in many studies, the high and clearly abundant FSHR expression at protein levels by immunohistochemistry or immunofluorescence was not associated with clear *FSHR* mRNA amplification (9, 11, 13). Nested polymerase chain reaction with gene-specific revers transcription, instead of traditional PCR, was needed to detect *FSHR* expression in umbilical cord, placenta, and uterus (9, 11, 13). This could indicate a short turnover time and/or rapid degradation of the *FSHR* transcripts or, not totally unlikely, nonspecific IHC staining results. Moreover, the lack of sequencing data of PCR products reduces the reliability of mRNA detection (9, 11, 13). In normal testis, there is ~0.04 pg of *FSHR* transcripts per μg total RNA (43), whereas human ovary contains a small amount of endogenous FSHR protein (0.054 fmol/mg of total protein) (44), and thus its detection by Western Blot might be challenging (45). One explanation could be that *FSHR* mRNA turnover in extragonadal tissues is much faster than in gonads? Most likely not, considering that in mammals long half-life for RNAs is *t*_(1/2)_ ≥ 4 h (e.g., housekeeping genes) and a short half-life for RNAs is *t*_(1/2)_ < 4 h (regulatory genes); thus we should still be able to detect *FSHR* mRNA at a level relevant to protein levels (46).

## Anti-FSHR Antibodies

Antibodies are an essential tool for determining cellular protein localization and expression. However, the commercially available monoclonal and polyclonal anti-FSHR antibodies have been poorly or not at all validated. Basic information on selected popular FSHR antibodies is listed in Table 1A. The specificity of two frequently used antibodies, sc-7798 and sc-13935, from Santa Cruz Biotechnology, has been recently revisited (47). In a comprehensive study, the authors compared these antibodies with FSHR323 (44) and two potential therapeutic anti-hFSHRs Ylanthia® antibodies (Y010913, Y010916) for their suitability in IHC detection of FSHR expression in various tissues (47). Specificity of all antibodies was tested by their binding to native hFSHR from different sources and by IHC on paraffin-embedded Flp-In Chinese hamster ovary cells transfected with *FSHR* (47). Unfortunately, human ovary or testis tissues were not examined, which would have served as proper positive controls (47). The study showed that only the FSHR323 antibody was suitable for target validation of hFSHR in an IHC setting. Furthermore, the authors confirmed their earlier reports on specific overexpression of FSHR in peripheral tumor blood vessels but could not repeat the previously reported FSHR overexpression in ovarian and prostate cancer cells (24, 25, 33, 47).

**Table 1A:** Basic information on several commonly used FSHR antibodies for protein localization.

**Antibody**	**Catalog number**	**Description**	**Targeted part of the protein**	**Producer**
FSHR323		Mouse monoclonal	N-terminal extracellular domain of human FSHR	Commercially unavailable
FSHR190		Mouse monoclonal	N-terminal extracellular domain of human FSHR	Commercially unavailable
FSHR225		Mouse monoclonal	N-terminal extracellular domain of human FSHR	Commercially unavailable
FSHR18	CRL-2688	Mouse monoclonal	N-terminal extracellular domain of human FSHR	ATCC
Anti-FSHR	ab219312	Mouse monoclonal	Recombinant full length protein corresponding to human FSHR	Abcam
Anti-FSHR	LS-A4004	Rabbit polyclonal	N-terminal extracellular domain of human FSHR	LifeSpan BioSciences
FSHR H-190	sc-13935	Rabbit polyclonal	Raised against amino acids 1–190 of human FSHR	Santa Cruz Biotechnology
FSHR N-20	sc-7798	Goat polyclonal	Raised against a peptide mapping near the N-terminus of human FSHR	Santa Cruz Biotechnology
Anti-FSHR		Rabbit monoclonal		Chemicon
Anti-FSHR				Zymed
Anti-FSHR	LS-C120589	Rabbit polyclonal	Synthetic peptide from human FSHR. (AA Range: 278–327)	Lifespan Biosciences
Anti-FSHR	Y010913, Y010916	Anti-human	Two different peptides of the hFSHR extracellular domain (aa295–332)	Commercially unavailable

From a rational point of study design, testing the specificity of FSHR antibodies using (transfected) cancer cell lines or extragonadal tissues cannot be considered sufficient. The most suitable tissues for the validation of specificity of reproducibility of an anti-FSHR antibody should be the human ovary and testis, the only tissues with undisputable FSHR expression. IHC for FSHR should also be tested on FSHR-negative control tissues. The FSHR323 antibody (44), suggested to be the most specific and reproducible, unfortunately has the major disadvantage of not being available either from the laboratory of its origin or commercially. The hybridoma cell line producing FSHR323 antibodies was removed from the American Type Culture Collection selection shortly after the milestone paper on FSHR expression in TVECs (26) was published in 2010, which created a major obstacle for independent validation of the recent studies on non-gonadal FSHR expression (26). If the authors/owners of this FSHR323 antibody are confident of its efficacy in detection of FSHR, they should make it available for other investigators, e.g., by re-depositing the hybridoma cell line to ATCC, so that independent verification of their data would become possible.

## FSHR in Normal Extragonadal Tissues

Recent studies on FSHR localization have suggested its expression in many normal extragonadal tissues including umbilical vein, vessel smooth muscle cells (9), placenta and placental endothelial cells (12), fallopian tube (11), myometrium, endometrial stromal cells, endometrial glandular epithelium (16, 17), liver (48) bone osteoclasts (20, 49), and monocytes (50, 51) (Table 1B).

**Table 1B:** Summary of studies presenting FSHR expression and FSH action in extragonadal normal and malignant tissues.

**Type of tissue/tumor (*n* = number of samples analyzed)**	**FSHR expression detection method**	**FSHR localization**	**References**
HUVECs and umbilical cord	IHC—FSHR323 Ab, RT-PCR, functional analysis	HUVECs, umbilical cord vessel smooth muscle cells, Wharton's jelly.	(9)
HUVECs and umbilical cord	IHC—FSHR323 Ab, RNAscope hybridization, RT-PCR (TaqMan) and functional analysis	No FSHR transcripts in FSHR-positive UC vessels detected by FSHR323 Ab.	(10)
Placenta, uterus	IHC FSHR323 Ab	Endothelial cells.	(11)
Mouse placentas	IHC—FSHR323 Ab; functional studies	Placental cells, endothelial cells.	(12)
Myometrium (*n* = 5)	IHC—FSHR323 Ab; qPCR; functional analysis	Non-pregnant and pregnant term non-laboring myometrium.	(13)
Myometrium (*n* = 3)	IHC—FSHR323 Ab; RT-PCR	Endothelial cells of myometrial vessels and arterial smooth muscle, weak in myometrial muscle fibers of the non-pregnant myometrium. Strong in the endothelial cells, arterial smooth muscle and muscle fibers in pregnant myometrium.	(11)
Endometrium	IHC—FSHR323 Ab	Decidual layer of the pregnant uterus and in non-pregnant endometrium in glandular epithelium and stromal cells of proliferative and secretive phase.	(11)
Endometrium (*n* = 12)	IHC—anti-FSHR Ab (Santa Cruz Biotechnology—SCB); RT-PCR; functional analysis	Endometrial gland cells throughout the glandular epithelium.	(14)
Endometrium (*n* = 7–8)	IHC—anti-FSHR (Chemicon) RT-PCR	Epithelial and stromal cells in both endometrium phases.	(15)
Endometriosis (*n* = 122) including Ovarian endometrioma—OE (*n* = 70) Recto-vaginal endometriotic nodules -RVEN (*n* = 52) Control endometrium (*n* = 30)	IHC—FSHR323 Ab; RNAscope hybridization; RT-PCR; functional analysis	Glandular epithelium and stromal cells of the secretory endometrium and RVEN.	(16)
Endometriosis (*n* = 194)	IHC—FSHR323 Ab	Endothelial cells, endometriotic glandular epithelial cells and endometriotic stromal cells.	(17)
Endometriosis (*n* = 38)	IHC—FSHR323 Ab; qPCR; functional analysis	Epithelial and stromal cells of endometriotic lesions, endometrial blood vessels of ectopic endometriotic tissues.	(18)
Bone (*n* = 6–18 mice per group)	RT-PCR; functional analysis	No Fshr expression was detected in long bone or in isolated cultured mouse osteoblast or osteoclast.	(19)
Bone (human and mouse osteoclasts and mesenchymal stem cells); RAW264.7 and RAW-C3 cells	ICC—anti- FSHR (Zymed); WB; RT-PCR; functional analysis.	Membrane of osteoclast precursors and mature osteoclasts.	(20)
Bone (5 mice per group)	NIR-II imaging of *Fshr in vivo*; functional analysis	Mouse bones *in vivo*.	(21)
Adipose tissue (*n* = 4 mice per group); adipocytes derived from mesenchymal stem cells (MSC-ad); 3T3.L1 cells	IHC—anti-Fr Ab (LS-A4004); functional analysis	Inguinal and visceral of white adipose and brown adipose tissue.	(22)
Adipose tissue from human and mouse (10 mice per group); 3T3.L1 cells	IHC—FSHR (Abcam); WB; RT-PCR; functional analysis	Cell membrane of adipocytes.	(23)
Prostate cancer and prostate cancer cell lines: PC-3 and DU145	IHC, WB, Flow cytometry, and functional analysis	FSH and FSHR expression in prostate cancer cells, PC-3 and DU-145 cell lines.	(24)
Prostate cancer (*n* = 30), BPH (*n* = 15), normal prostate (*n* = 13). Prostate cancer cell lines PC-3 and LNCaP	RT-PCR, IHC, and functional analysis	High FSHR expression in prostate cancer cells. Lower FSHR expression in hyperplastic benign prostate and normal prostate.	(25)
Prostate (*n* = 773), breast (*n* = 112), colon (*n* = 15), pancreas (*n* = 67), bladder and kidney (*n* = 77), lung (*n* = 15), liver (*n* = 15), stomach (*n* = 6), testis (*n* = 8), ovary (*n* = 6)	IHC—FSHR323 Ab, FSHR190 Ab, FSHR225; FISH—NA	High clear FSHR expression in TVECs. No expression in normal and inflammatory tissues.	(26)
Kidney cancer (*n* = 50)	IHC—FSHR323 Ab	TVECs	(27)
Primary tumors and metastases: prostate (*n* = 76), lung (*n* = 46), breast (*n* = 42) colon (*n* = 34), kidney (*n* = 5)	IHC—FSHR323 Ab	TVECs	(28)
Breast cancer (n = 83)	IHC—FSHR323 Ab	TVECs	(29)
Ovarian tumors (EOC, *n* = 156)	IHC, Ab NA	FSHR is expressed in ovarian tumors (64.3% analyzed samples).	(30)
Ovarian tumors (EOC, *n* = 153)	IHC, Ab NA	Her-2 can be a negative prognosticator only in FSHR negative EOC cases.	(31)
Ovarian cancer—*in vitro* study	Functional studies on HO8910 and HEY cell lines	FSH induced the epithelial-mesenchymal transition of ovarian cancer cells through the FSHR-PI3K/Akt-Snail signaling pathway.	(32)
Ovarian cancer (*n* = 77)	IHC, FSHR18 Ab, WB, FSHR18 Ab, qPCR, TaqMan FSHR probe	Gynecologic malignancies of different histological types, but not in non-ovarian healthy tissues.	(33)
Primary rhabdomyosarcoma (*n* = 58)	RT-PCR; functional analysis *in vitro*	Rhabdomyosarcoma cell lines and primary lesions.	(34)
Neuroendocrine tumors (*n* = 14)	IHC—FSHR (sc-13935), SCB	50% of TVECs and majority of tumor cells.	(35)
Pancreatic neuroendocrine tumors (*n* = 30)	IHC, IF and WB with FSHR (H-190), FSHR (N-20), FSHR (N-20) Ab from SCB.	Neoplastic cells but no expression in TVECs.	(36)
Thyroid neoplasms (*n* = 44)	IHC—FSHR (sc-13935) SCB	Cancer cells and TVECs.	(37)
Thyroid neoplasms (*n* = 36)	IHC—FSHR (sc-13935) SCB	Cancer cells and TVECs.	(38)
Thyroid neoplasms (*n* = 312)	IHC, Ab NA	Normal and neoplastic thyroid epithelial cells except undifferentiated carcinoma.	(39)
Pituitary adenomas (*n* = 42)	IHC—FSHR (sc-13935) SCB	Adenoma cells and TVECs.	(40)
Pituitary adenomas (*n* = 28)Adrenal tumors (*n* = 36)	IHC—FSHR (sc-13935) SCB	Adenoma cells and TVECs.	(41)
Soft tissue sarcomas (*n* = 335, 11 subtypes)	IHC FSHR323	TVECs and tumor cells.	(42)

*IHC, immunohistochemistry; NA, not applicable; UC, umbilical cord; TVECs, tumor vessel endothelial cells*.

## Placenta, Umbilical Cord Vessels, and Human Umbilical Vein Endothelial Cells (HUVECs)

Human placenta and umbilical cord express FSHR, and these reports proposed that the expression is functional (details below) (9, 11). FSHR was found in the endothelial cells of the fetal vasculature within the chorionic villi and villous stromal cells from 8 to 10 week of gestation until term, but not in trophoblast cells (11). In human umbilical cord, the location of FSHR was suggested in Wharton's jelly, and endothelial and smooth muscle cells of the vessels (9). Expression of a splice variant lacking exon 9 was detected in primary HUVECs (9).

Further functional studies showed that FSH-stimulated HUVECs responded with AKT activation, but not with cAMP production (9). HUVECs stimulated with a high pharmacological dose of rhFSH (600 μg/L, equals to 81.86 IU/L) induced significantly tube formation, wound healing, cell migration and proliferation, nitric oxide production and cell survival (9). This prompted the authors to propose that HUVECs express “functional” FSHR expression and that the FSH-FSHR activation promotes angiogenesis as effectively as the potent well-characterized angiogenic factor VEGF (9). However, opposite results were published by our group upon revisiting the same study concept and experiments (10). In this latter study, neither FSHR expression nor rhFSH stimulation of freshly isolated HUVECs could be shown (10). In our study, *FSHR* expression was analyzed with two kits for reverse transcription and qPCR systems, and both gave negative results for HUVECs and an immortalized HUVEC cell line (HUV-ST) (10). The PCR products from the positive control samples were sequenced in order to reconfirm their fidelity, which was not done in the earlier study (9). Numerous methodological uncertainties [presented in detail in (10)], made the results of the previous study hard to interpret and could explain the difference between these two studies (10). Consequently, our data (10) do not support the novel concept that FSH-FSHR activation is involved in the placental vasculature and its angiogenic process (11–13). Results showing that *Fshr/FSHR* is essential for normal angiogenesis of the fetal placental vasculature (12) are also contradictory because both males and females with inactivating *FSHR* mutations, thus obligatorily devoid of functional FSHR in their placenta, appear to develop normally *in utero* (52). Thus, caution is needed to interpret the existing data on the FSH-FSHR activation in placental vasculature and their proangiogenic process, and the functional expression of FSHR in HUVECs still remains a subject of uncertainty.

## Uterus (Endometrium and Myometrium)

Several studies suggest that FSHR is expressed in uterine tissues (11, 15–18, 53). One study appeared to detect positive FSHR signal in the endothelial cells of myometrium vessels and arterial smooth muscle of the normal non-pregnant myometrium, whereas signal in myometrial muscle fibers was weak or only traceable (11). Furthermore, FSHR staining was detected in the endothelial cells, arterial smooth muscle and muscle fibers of myometrium during pregnancy (11); however, the study group was small (*n* = 3). They also showed through PCR analysis that human myometrium from term pregnancy expresses full-length FSHR mRNA, but the PCR product were not sequenced to reconfirm their fidelity (11). In another study, in term pregnancy non-laboring myometrium, FSH increased contractile activity, but the effects was observed only at supraphysiological doses of 100–1,000 μg/L (136.43–1364.25 IU/L) (13). In contrast to findings of the former study (11), another recent study did not detect FSHR protein or *FSHR* transcripts in normal human myometrium (16).

Intriguingly, women with inactivating *FSHR* mutation, thus lacking both gonadal and extragondal FSHR, can maintain normal pregnancy until term following ovum donation, thus undermining the functional importance of extragonadal FSH action (54).

In human endometrial epithelial and stromal cells, cycle-dependent expression of FSHR was shown, and FSHR expression increased during the endometrial secretory phase (15). Furthermore, it has been shown that FSH stimulation induced a decidual phenotype in stromal cells isolated from proliferative phase human endometrium (53). Microarray studies also proposed that FSHR mRNA expression was up-regulated in human endometrial stromal cells decidualized with progesterone and cAMP (55). Moreover, results of our study showed that FSH stimulation upregulated *FSHR* expression in human endometrial stromal cells undergoing decidualization *in vitro* (16). It has been shown that during pregnancy, FSHR is expressed in the decidual layer of the pregnant uterus in women (11). FSHR was localized in glandular epithelium and stromal cells of endometrial proliferative and secretive phases. A weak, mainly basolateral FSHR expression was localized in the microvascular endothelium of the normal proliferative endometrium (17). Our group confirmed FSHR expression at mRNA and protein levels in glandular epithelium and stromal cells of normal human secretive endometrium, but not in the proliferative endometrium (16). A very recent study also proposed the functionality of FSHR expression in human endometrium, as endometrial tissue produced cAMP upon FSHR stimulation (14). It seems that FSHR expression in human endometrium, mainly in secretive phase could be functional, but the concept that FSH/FSHR signaling may induce human endometrial stromal cell decidualization requires further evidence.

## Adipose Tissue/Obesity

The rise of FSH level in menopausal women is associated with increased visceral adiposity, and decreased bone density and energy expenditure (56). In human and mouse fat tissues and adipocytes, FSHR expression at mRNA and protein levels has been reported (23). Recently, Liu et al. published that blockage of FSH signaling with a specific polyclonal FSH antibody potentially activated brown/beige fat thermogenesis and thereafter reduced the total fat, subcutaneous fat and visceral fat volume induced by high-fat diet in wild-type mice (22). This antibody also reduced adiposity in ovariectomized mice. Immunostaining analysis showed the presence of FSHR in inguinal and visceral white adipose tissues and also in brown adipose tissues in mice. However, this study lacks the proper negative and positive (testis or ovary) tissue controls to confirm the specificity of the used FSHR antibody. To reconfirm the *Fshr* expression in adipocytes, the authors also sequenced full-length *Fshr* cDNA from primary mouse mesenchymal stem cells derived from ear lobes and mouse adipocyte-like 3T3.L1 cells (22). However, even though the expression profile of many genes was shown in white and brown adipose tissue in mice in this study, surprisingly no data on *Fshr* at mRNA levels was reported in those tissues. For proper FSHR expression assessment, the same tissues/cells should be used for both gene expression and protein analysis. Moreover, they showed by immunoprecipitation assay that their established FSH antibody binds to FSHβ, but the study lacks data on direct evidence for FSH binding to adipocyte FSHR (22). Another group of treatment with GnRH antagonists to block the gonadotropins (FSH) would have strengthened the findings. The most intriguing part of this FSH antibody-based data is that it contradicts with data from mice and humans using GnRH agonists/antagonists, where the endpoint is the same—blockage of gonadotropins. We did not observe any loss of body fat in transgenic mice treated by GnRH antagonist in conjunction with gonadal or adrenocortical tumor treatment (57, 58). FSH suppression concomitant with that of gonadal steroids did not decrease body fat in a clinical study on men (59). This group (22) has developed now an HF2 monoclonal antibody that targets human FSHβ, but no reports in humans have been shown yet (60).

## Bone Mass/Osteoporosis

It has been proposed that enhanced postmenopausal bone resorption is caused not by estrogen deficiency, but by increased FSH level (61). Direct effect of FSH-FHSR on bone loss in mice has recently been shown (20–22). FSHR was localized in mouse and human osteoclasts and mesenchymal stem cells (20, 62). The latest study reported that blockage of FSH action by a specific monoclonal antibody targeting FSHβ subunit increased bone mass and stimulated new bone formation by osteoblasts in mice (22). Moreover, osteoclastogenesis and expression of osteoclast-specific genes, and matrix metalloproteinase-9, were reduced after attenuation of FSH action (21). The authors showed the presence of FSHR in mouse bones only *in vivo*, through a specific binding of the fluorescently labeled FSH with near-infrared II fluorophore to FSHR (21). They did not perform any qPCR, immunohistochemistry or Western Blot analysis to demonstrate and/or directly localize FSHR expression in bone. Furthermore, as in the study of FSH action in adipocytes, there is no data showing direct FSH binding to FSHR in bone cells (21, 22).

Human studies are not able to replicate the data from the mouse models mentioned above. A direct interventional study showed that FSH does not regulate bone loss in postmenopausal women (63). Suppression of FSH secretion by GnRH agonist did not decrease the level of bone resorption markers, but rather tended to increase some of them (63). In addition, in normal adult men, FSH did not affect bone turnover and thus appears not to be an important regulator of skeletal metabolism (64). Another *in vivo* study in men showed that sex steroids modulate bone resorption independently of FSH action (65). Blockage of androgen action by GnRH agonist treatment in prostate cancer patients induced increased fracture risk and bone loss, with no positive effect of FSH suppression (66). Moreover, also some mouse studies showed opposite results (19, 67), in contrast to the earlier study (20). Furthermore, FSHR knockout mice have reduced bone mass (20, 67), and a dose-dependent FSH effects on increased bone mass through an ovary-dependent mechanism in female mice have been observed (19). In male mice FSH impact on bone loss has not been demonstrated (68). Taken together, further studies are needed to determine whether the FSH-FSHR system action in bone resorption is really functional, only species-specific, a probable indirect effect of inflammatory cytokines such as tumor necrosis factor α (TNF-α) (69) or an unidentified off-target effect of the FSH antibodies used.

## Endometriosis

Expression of FSHR has also been suggested in endometriosis (16–18). An immunohistochemical study described FSHR localization in the endometriotic glandular and stromal cells of the rectovaginal endometriotic nodules, ovarian endometriotic cysts, and peritoneal endometriotic lesions, as well as with robust vascular FSHR staining (17). Recently, our group showed functional FSHR expression in deep endometriotic lesions, where FSH through FSHR locally up-regulated aromatase expression and induced estrogen production, and FSHR localized at mRNA and protein levels in endometriotic glandular and stromal cells (16). Moreover, the ovarian pattern of differentiation with functional FSH-FSHR system, inducing production of the same steroidogenic cascade as in ovaries has been shown in endometriotic tissues (18) Based on these results, it appears that the expression of FSHR in endometriosis is functional (16, 18).

## FSHR in Cancer and Tumor Vessel Endothelial Cells (TVECs)

A number of studies (summarized in Table 1B) have shown the expression of FSHR in different types of tumor cells suggesting a potential role of FSH in tumorigenesis and suggesting FSHR as a potential target for cancer therapy. FSHR expression was suggested to be present in prostate, ovarian, thyroid, neuroendocrine pancreatic, and pituitary cancer cells and soft tissue sarcomas (Table 1B). A breakthrough in the study of extragonadal FSHR expression was, when the receptor expression was proposed in TVECs of different types of tumors (prostate, breast, colon, pancreas, urinary bladder, kidney, lung, liver, stomach, testis, and ovary), including their metastases (26, 27, 29). In the prostate cancer FSHR-positive arterioles, capillaries and venules (but not lymphatic vessels) were located at the periphery of the tumor core (26). Endothelial cells of the vessels present in normal-appearing tissues at a distance >10 mm outside the tumors or normal prostate were FSHR-negative (26). In subsequent studies, endothelial FSHR expression was shown in thyroid and neuroendocrine tumors (35, 37, 40). Several groups suggested that the FSHR observed in TVECs and cancer cells is functional (24–26, 70–72). FSH-stimulated proliferation, migration and invasion was observed in epithelial cancer cells (70). In TUVECs, FSHR mediated the FSH transport, tumor angiogenesis and vascular remodeling (26, 29). With regard to cancer cells and TVECs (primary and metastatic), it was suggested that FSHR might serve as a potential cellular marker of different tumors and provide a novel approach for targeted cancer therapy. If extragonadal FSHR expression and function could be proven by additional independent studies in the TVECs, as well as in the cancer cells, the finding would suggest for FSHR a great utility as a biomarker and/or medicinal target. This potentially important finding needs further verification by independent studies.

## Proposals to Improve the Quality of FSHR Localization—Validation of Anti-FSHR Antibodies

In light of the information reviewed above, it seems that the data on extragondal FSHR localization often relies on insufficiently verified antibodies, making interpretation of data difficult; more worryingly, to potentially erroneous conclusions. As an additional validation of FSHR IHC, we propose the use of RNAscope *in situ* hybridization (73) or other similar methods. This novel and sensitive method displays a single-transcript resolution and is now in common use. A set of validated, negative, and positive control probes (for genes with different expression levels), provided by manufacturer confirm the quality of the tissue used for hybridization. Using the FSHR323 antibody (donated to us by Dr. Ghinea), we were able to confirm FSHR expression in umbilical vein endothelial cells at protein level but not *by in situ* hybridization or qPCR on mRNA levels, leaving the question open why the expression at protein level is not supported by mRNA data (10). Recently it has been shown that endothelial cells and smooth muscle cells of umbilical cord vessels, despite clear FSHR immunoreactivity using FSHR323 antibody (9), did not express *FSHR* transcripts, even when confirmed by a gel electrophoresis of qPCR products (10). A similar discrepancy was observed in endometriosis samples where FSHR at protein level has been shown in endothelial cells of the endometroid tissue (17), but could not be reproduced by *in situ* hybridization at mRNA level (16).

## *In vivo* and *in vitro* Models to Study Extragonadal FSH Action

To resolve the controversies of direct FSH action in extragonadal tissues proper *in vivo* mouse models would be very useful, as well as functional testing on the extragonadal receptors *in vitro*. *Fshr* null mice would be a good model for such studies; although these mice exhibit extragonadal defects, data from different scientific groups show contradictory results (20, 67). *In vitro* experiments on primary cell cultures from extragonadal tissues with purported FSHR expression have also yielded contradictory data (9, 10). Some *in vitro* studies could be performed on extragonadal tissue explants, allowing to examine mechanisms without disturbing tissue structure and tissue receptor status (16). However, to explain all inconsistencies *in vivo* and to prove that the extragonadal FSH-FSHR system is really functional in different extragonadal tissues, its actions should be evaluated through precise gene targeting, e.g., using the genome-editing CRISPR/Cas9–based method (74). Conditional knockout mouse models with tissue-specific deletion of FSHR *via* Cre-lox technology would allow us to directly analyze the loss of FSH-FSHR action in desired condition or extragonadal tissue type. A transgenic mouse model expressing human FSHR with a green fluorescent protein (GFP) or any other reporter marker expression under the FSHR promoter/enhancer could be also a very useful tool to track the FSHR expression. Thereafter, all data can be tested *in vitro* and *in vivo* with the same system to prove their functionality.

## Gender Differences Putatively Affecting the Extragonadal FSHR Expression

Many of the studies on extragonadal FSHR function were carried out only on women (endometrium, endometriosis, HUVECs etc.) (9, 10, 12, 17, 18) or female mice (osteoporosis, bone metabolism, or the FSH action on the myometrium muscle contractions etc.) (11, 13, 21, 22, 63) or cells derived from females (for example HUVECs) (9, 10, 12). Almost the only exception are the human prostate cancer tumor vessel cells or human prostate cancer tissue/cells (24–26). Some studies do not state properly the sex of the individual or sample. One cannot rule out the option that some pathologies (for example osteoporosis or bone metabolism etc.) have strong sex differences and are influenced differently by sex steroids and in general the sexually dimorphic endocrine functions.

## Conclusions

FSHR expression in extragonadal tissues, despite numerous publications, remains controversial. We suggest a more critical analysis of such data, especially when the localization of FSHR in normal extragonadal and malignant tissues is based only on IHC data, without additional methodological confirmation. We suggest proper validation of antibody specificity and the reproducibility between lots. Unrestricted access to the currently existing non-commercial anti-FSHR antibodies would support the fairness of science. When planning experiments, more attention should be paid to inclusion of proper positive and negative controls. For functional studies, the doses of rhFSH should be standardized and restricted to physiologically meaningful concentrations. A proof of principle in human clinical trial with the monoclonal HF2 FSH blocking antibody with a comparison to a group of patients with GnRH antagonist is also needed. It is thus probably not yet time to re-write the textbooks with the functional implications of FSH/FSHR in extragonadal tissues or in cancer cells or TVECs. To this end, definitely many more studies are needed.

## Author Contributions

MC, DP-T, SW, IH, and NR took part in designing, drafting, and finalizing of this invited review manuscript.

### Conflict of Interest Statement

The authors declare that the research was conducted in the absence of any commercial or financial relationships that could be construed as a potential conflict of interest.
